# Effects and Management of *Parthenium hysterophorus*: A Weed of Global Significance

**DOI:** 10.1155/2014/368647

**Published:** 2014-08-20

**Authors:** Manpreet Kaur, Neeraj Kumar Aggarwal, Vikas Kumar, Romika Dhiman

**Affiliations:** Kurukshetra University, Kurukshetra, Haryana 136119, India

## Abstract

Congress grass, *Parthenium hysterophorus* L., of the family Asteraceae (tribe: Heliantheae), is an erect and much branched annual or ephermeral herb, known for its notorious role as environmental, medical, and agricultural hazards. It is believed to have been introduced into India and Australia from North America and in the last few years the weed has emerged as the seventh most devastating weed in Africa, Asia, and Australia. The aim of this review is to provide general information about the physiology, distribution, ill effects, and management of parthenium. Control of parthenium has been tried by various methods, but no single management option would be adequate to manage parthenium, and there is a need to integrate various management options. Successful management of this weed can only be achieved by an integrated approach with biological control as the key element.

## 1. Introduction

*Parthenium hysterophorus *L. (Asteraceae), a noxious plant, inhabits many parts of the world, in addition to its native range in North and South America and the West Indies [1]. According to Holm et al. [2] this noxious invasive species is considered to be one of the worst weeds currently known. This is a weed of global significance responsible for severe human and animal health issues, such as dermatitis, asthma and bronchitis, and agricultural losses besides a great problem for biodiversity. It is a widely held belief that the seeds of this weed came to India with grains imported from USA under the US PL 480 scheme, also known as “Food for Peace” which is a food assistance programme of the US government, and spread alarmingly like a wild blaze to almost all the states in India and were established as a naturalized weed. In India, the weed was first pointed out in Poona (Maharashtra) by Professor Paranjape, 1951, as stray plants on rubbish heaps and was reported by Rao [3] as a new species in India, but the earliest record of this species in India goes back to 1814 by Roxburgh, the father of Indian Botany, in his book* Hortus Bengalensis* [3, 4]. Ever since the weed became a menace around the globe including India, efforts have been made to manage the weed employing different methods such as mechanical, competitive replacement (allelopathy), chemical, and biological control methods. However, the weed has defied all human efforts to control it due to one or other disadvantages. Biological control, the intentional manipulation of natural enemies, insects, bioherbicides, nematodes, snails, and competitive plants to control harmful weeds, is gaining momentum as it is an effective and ecofriendly alternative to conventional methods of weed control [5].

## 2. Distribution and Biology of Parthenium Weed

### 2.1. Distribution

Parthenium is native to the area surrounding the Gulf of Mexico, Central America, southern North America, West Indies, and central South America [1, 6]. The weed has now invaded more than 20 countries around the globe, including five continents and numerous islands. Recent developments have indicated that African countries are at high risk of invasion. It is now also present in eight provinces of China and spreading at an alarming rate. Partheniumprobably entered India before 1910 (through contaminated cereal grain) but went unrecorded until 1956. Since 1956, the weed has spread like wildfire throughout India [7].

### 2.2. Name

The genus name Parthenium is derived from the Latin word parthenice—a reference to the plant now known as* Tanacetum parthenium* (L.) Bernh. or “feverfew;”* hysterophorus* was derived from the Greek* hystera *(womb) and* phoros* (bearing), referring to the prolific seeding habit of the plant [8]. It is commonly called as bitter weed, carrot weed, broom bush, and congress grass (India); whitetop, escobar amarga, and feverfew (Caribbean) and; false ragweed and ragweed parthenium (USA).* Parthenium hysterophorus* L. (parthenium weed) is a member of the tribe Heliantheae of the family Asteraceae, an extremely diverse family with a cosmopolitan distribution [6].

### 2.3. Morphology of the Plant

**Figure 1:**
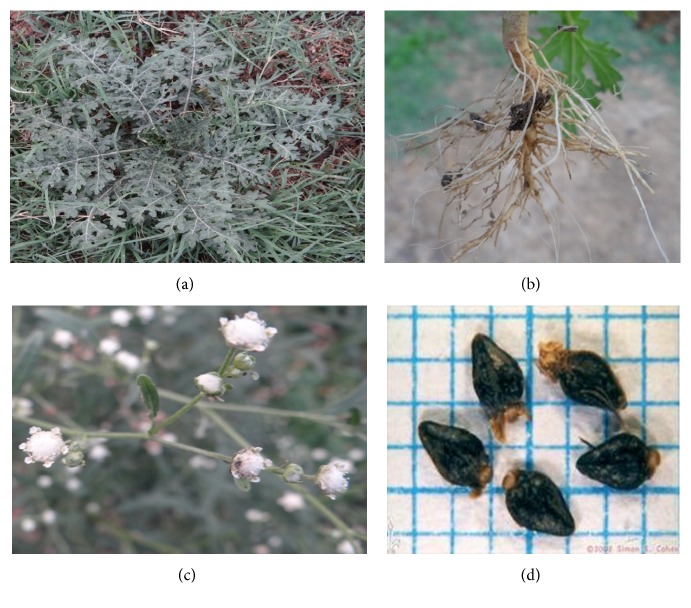
Parthenium weed; (a) rosette stage of parthenium plant; (b) tap root system of parthenium;(c) capitula; and (d) black wedge shaped seeds.

*P. hysterophorus* L. of the family Asteraceae (tribe: Heliantheae) is fast maturing, erect, and much branched annual or ephemeral herb. It shows two distinct phases in life: juvenile, rosette, or the vegetative stage and adult, mature, or the reproductive stage. The juvenile stage exhibits a rosette with large, dark green, simple, radicle, and pinnatisect small leaves lacking flowering (Figure 1(a)). The large lower leaves are spread on the ground like a carpet, without allowing any vegetation underneath it [9]. The adult stage is erect, much branched with deep tap root system that reaches up to 2 m in height (Figure 1(b)). The stem is hairy, octangular, longitudinally grooved and becomes tough and woody as the plant matures into a hardy bush. Leaves are simple, alternate, pinnately or bipinnately dissected (Figure 1(a)), 20–30 × 12–25 cm, becoming smaller towards the apex of the branches. The stem and leaf surface is covered with four types of glandular and nonglandular, multicellular white trichomes. The flowers are creamy white, about 4 mm across, arising from the leaf forks. Enormous number of pollen grains, 624 millions/plant, are produced which are anemophilous, that is, wind pollinated. Each flower produces four to five black wedge shaped seeds (Figures 1(c) and 1(d)) that are 2 mm long with thin white scales and difficult to see by the naked eye. It is a very prolific seed producer, producing up to 25,000 seeds/plant, leading to large seed bank in the soil [10].

### 2.4. Habitat

**Figure 2:**
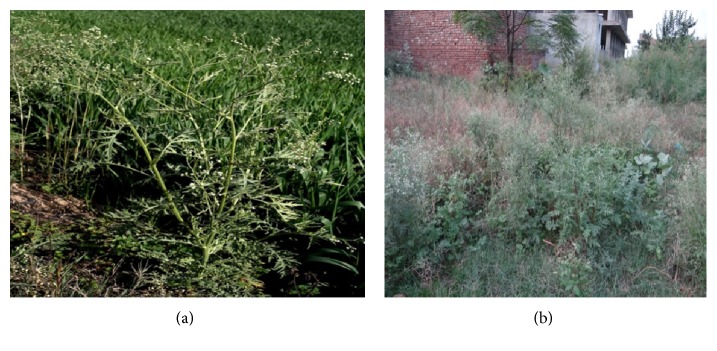
Area of infestation of parthenium; (a) crop field infestation; (b) residential plot infestation.

Parthenium grows luxuriantly in wastelands, public lawns, orchards, forestlands, flood plains, agricultural areas (Figure 2(a)), urban areas, overgrazed pastures, industrial areas, playgrounds, roadsides, railway tracks, and residential plots (Figure 2(b)). Drought and subsequent reduced pasture cover create the ideal situation for the parthenium weed to establish itself. Although parthenium weed is capable of growing in most soil types, it is most dominant in alkaline, clay loam soils.

### 2.5. Dispersal and Germination of Seeds

The seeds are mainly dispersed through water currents, animals, movement of vehicles, machinery, grains, stock feed and to a lesser extent by the wind. Most of the long distance spread is through vehicles, farm machinery, and flooding. The spread of seeds plus their ability to remain viable in the soil for many years pose one of the most complex problems for control [11]. Seeds do not have a dormancy period and are capable of germinating anytime when moisture is available. Seeds germinate within a week with the onset of monsoon and flowering starts after a month and continues up to another three months. In northwest India, parthenium germinates mainly in the months of February-March, attaining peak growth after rains in June-July and produces seeds in September-October. It normally completes its life cycle within 180–240 days. Its growth remains less and stunted from November to January due to severe cold [7, 12].

## 3. Harmful Effects

Parthenium is considered as the number one dangerous terrestrial weed because of its harmful effects both to humans and to biodiversity which are discussed below.

### 3.1. Effects on Ecosystem

Parthenium has been reported to be causing a total habitat change in native Australian grasslands, open woodlands, river banks, and flood plains [9]. It is an aggressive colonizer of wasteland, road sides, railway sides, water courses, cultivated fields, and overgrazed pastures and has invaded 14.25 million hectares of farm land during 2001–2007, compared to 2 million hectares in 1991–2000 [10].

### 3.2. Effects on Crops

Parthenium plant contains chemicals, like parthenin, hysterin, hymenin, and ambrosin, and due to the presence of these chemicals, the weed exerts strong allelopathic effects on different crops. Parthenin has been reported as a germination and radical growth inhibitor in a variety of dicot and monocot plants [13]. The weed affects nodulation in legumes due to inhibition of activity of nitrogen fixing and nitrifying bacteria, namely,* Rhizobium, Actinomycetes, Azotobacter*, and* Azospirillum. *Parthenium produces enormous numbers of pollens (on an average 624 million/plant), which are carried away at least to short distance in clusters of 600–800 grains, and settles on the vegetative and floral parts, including stigmatic surface, inhibiting fruit setting in crops like tomato, brinjal, beans, capsicum, and maize. In India,* P. hysterophorus* causes a yield decline of up to 40% in agricultural crops, Khosla and Sobti [14]. Sorghum (*Sorghum bicolor *L. Moench) grain yield losses between 40 and 97% have been reported in Ethiopia if parthenium is left uncontrolled throughout the season [7, 15]. In Australia,* P. hysterophorus* infests around 170000 km^2^ of prime grazing country in Queensland, causing economic losses of around $16.8 million per year to the pasture industry [16]. On cracking clay soils with an annual rainfall between 600 and 800 mm,* P. hysterophorus *was estimated to reduce the carrying capacity of affected farms in Australia by about 40% [17, 18]. The weed also acts as a collateral host for many diseases caused by viruses in crop plants.

### 3.3. Effects on Animals

Parthenium weed is toxic to animals causing dermatitis with pronounced skin lesions on various animals including horses and cattles. If eaten, it is responsible for mouth ulcers with excessive salivation. Significant amount (10–50%) of this weed in the diet can kill cattle [19]. In addition, it causes anorexia, pruritus, alopecia, diarrhea, and eye irritation in dogs. It also causes acute illness, when bittermilk and tainted meat from buffaloes, cows and goats, are fed on grass mixed with parthenium [12]. The parthenium extract results in significant reduction of rat WBC count which signifies its immune system weakening ability [20].

### 3.4. Effects on Human Beings

The pollen grains, airborne dried plant parts, and roots of parthenium cause various allergies like contact dermatitis, hay fever, asthma, and bronchitis in human beings. The common allergens found in this weed are parthenin, coronopilin, tetraneuris, and ambrosin. Pollens of parthenium cause asthma (allergic bronchitis), especially in children playing outdoors and in adults and old-age persons. Contact of plant with the body causes dermatitis and the spread of the problem all over the body causes great discomfort [21]. Clinically the parthenium dermatitis is of five types, as discussed below.

**Figure 3:**
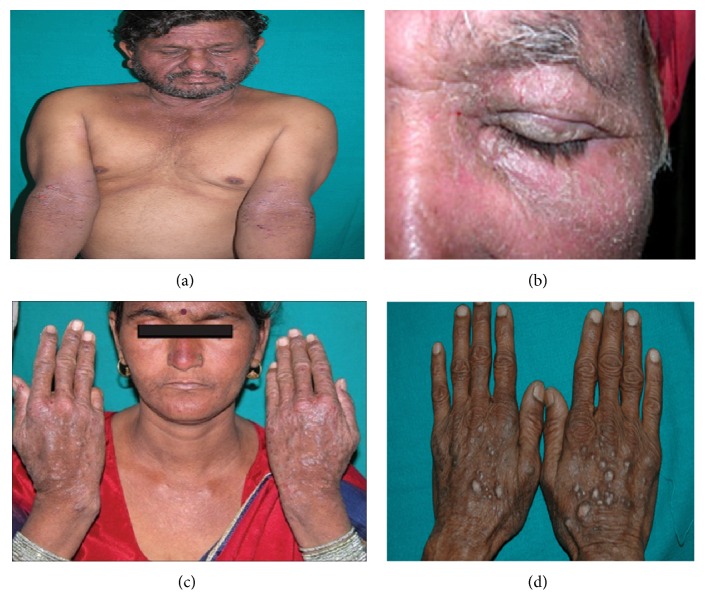
Four of the five types ofsymptoms of commonly known parthenium dermatitis; (a, b) airborne contact dermatitis; (c) chronic actinic dermatitis in a female; and (d) prurigo-like lesions over dorsa of hands.

(1) The classical pattern also known as* airborne contact dermatitis (ABCD)* (Figures 3(a) and 3(b)) affects the face, especially eyelids and/or neck, V of chest, cubital, and popliteal fossae; (2) the* chronic actinic dermatitis (CAD)* (Figure 3(c)) pattern involves the exposed areas such as forehead, rim of ears, cheeks, nape of neck, dorsae of forearms, and hands as lichenified papules, plaques, or papulonodules with relative sparing of nonsun exposed areas such as eyelids, retroauricular areas and undersurface of chin and depth of the skin folds; (3) the* mixed pattern* (combination of classical and CAD pattern) manifests as scattered infiltrated scaly papules over the exposed parts and dermatitis over eyelids, flexures of extremities and neck; (4) the* photosensitive lichenoid eruption pattern* presents with pruritic, discrete, flat, violaceous papules, and plaques over sun-exposed parts such as forehead, ears, cheek, upper chest, and back, extensor aspect of forearms and dorsae of hands stimulating photosensitive lichenoid eruptions; (5) and the* prurigo nodularis-like pattern* presents as multiple hyperkeratotic papules and nodules over extremity with characteristic histopathologic features similar to prurigo nodularis (Figure 3(d)) [12, 22].

## 4. Control of Parthenium

Singh (1997) considered use of biocontrol agents (insects and fungal pathogens) and exploitation of competitive plants (allelopathy), the most economic and practical way of managing parthenium. But the weed has not been managed below the threshold level and is threatening biodiversity and posing ill problems for the humanity and animals. Various methods, for example, physical, chemical, bioherbicidal, and integrated, are being practiced to manage this weed around the globe and are discussed.

### 4.1. Physical Control

Manual uprooting of parthenium before flowering and seed setting is the most effective method. Uprooting the weed after seed setting will increase the area of infestation. Some landholders have achieved success in ploughing the parthenium weed in the rosette stage before it seeds, but this must be followed up by sowing a crop or direct seeding the perennial pasture. Physical control involves hand weeding, a time consuming and unpleasant job, made worse by the health hazards involved with handling parthenium weed.

Burning, another strategy employed to manage weed, is not a useful control strategy for parthenium. However, research suggests that burning for other purposes (e.g., woody weed control) will not result in an increased infestation of parthenium as long as the pasture is allowed to recover before stock is introduced. This too has proved to be inadequate due to two reasons; it requires large quantity of fuel and burning destroys all other economically important plants growing in its vicinity [23, 24].

### 4.2. Chemical Control

Chemical control is an effective method to control parthenium in the areas where its natural enemies are absent. Use of chemical herbicides, such as chlorimuron ethyl, glyphosate, atrazine, ametryn, bromoxynil, and metsulfuron, are known to be very effective in controlling this weed. References [25–27] reported that the application of 2,4-D EE (0.2%) and metribuzin (0.25 and 0.50%) were found more effective for controlling parthenium at 15 days after spraying (DAS), causing complete kill of parthenium population, and did not allow any emergence of weed. Khan et al. [28] reported that the stage/time of parthenium weed for herbicidal control is important and the weed was effectively controlled at rosette stage in wasteland, noncropped areas, along railway tracks, water channels, and roadsides. The most effective treatments for parthenium weed control were glyphosate and metribuzin, having higher mortality at 4 weeks after treatment (WAT) at both rosette and bolted stages than 2, 4-D, triasulfuron + terbutryn, bromoxynil + MCPA and atrazine + s-metolachlor, atrazine, s-metolachlor. Pendimethalin was the least effective treatment for both growth stages. Overall, the efficacy of herbicides was promising on rosette parthenium plants than bolted plants. The mortality rate by different herbicides at rosette and bolted stages is given in Table 1. In open wasteland, noncropped areas and along railway tracks and roadsides, the spraying of a solution of common salt (Sodium chloride) at 15–20% concentration has been found to be effective.

#### 4.2.1. Disadvantages of Herbicides

There are several disadvantages of using the chemical herbicides, such as the environmental hazards and the development of resistance against many herbicides, like atrazine 2, 4-D, metribuzin, paraquat (Gramoxone), trifluralin, diphenamid, and glyphosate [29–31]. Glyphosate is one of the most toxic herbicides, with many species of wild plants being damaged or killed by applications of less than 10 micrograms per plant. Moreover, glyphosate can be more damaging to wild flora than many other herbicides. Atrazine has been found to be highly persistent in soil and has been classified as a restricted use pesticide (RUP) in the USA due to its potential for groundwater contamination [32].

### 4.3. Allelopathic Control

The term allelopathy was coined by Molisch (1937), which generally refers to the detrimental effect of one plant species on seed germination, growth, and reproduction of another plant species. Numerous plants are reported to possess allelopathic potential and efforts have been made to use them in weed control [33]. Competitive replacement of parthenium can be achieved by planting plants like* Cassia sericea, C. tora, C. auriculata, Croton bonplandianum, Amaranthus spinosus, Tephrosia purpurea, Hyptis suaveolens, Sida spinosa, *and* Mirabilis jalapa* which are capable of effectively suppressing partheniumin natural habitats [34]. A study in India revealed that* Cassia sericea* reduces the accumulation of parthenium by 70% and parthenium population by 52.5% [35]. Another study showed that aqueous extracts from* Imperata cylindrica*,* Desmostachya bipinnata, Otcantium annulatum,* and* Sorghum halepense* markedly suppressed seedling growth and germination of parthenium [36]. In India, crop rotation using Marigold (*Tagetes* spp.) during the rainy season, instead of the usual crop, has been found effective in reducing parthenium infestation in cultivated areas.

Both the root and shoot extracts of three allelopathic grasses, namely,* Dicanthium annulatum*,* Cenchrus pennisetiformis,* and* Sorghum halepense*, reduce germination and suppress early seedling growth of exotic weed* P. hysterophorus. *Aqueous foliar extracts of* Azadirachta indica, Aegle marmelos, *and* Eucalyptus tereticornis *totally inhibited the seed germination of partheniumand may be exploited for controlling parthenium weed.

### 4.4. Biological Control

Biological control is an environmentally sound and effective means of reducing or mitigating pests and pest effects through the use of natural enemies. In the last three to four decades, a great deal of emphasis has been given to control parthenium through various biocontrol agents like microbial pathogens, insects, and botanicals [24, 37]. Of the various biocontrol strategies, biological control of weeds by plant pathogens has gained acceptance as a practical, safe, and environmentally beneficial method applicable to agroecosystem [38]. There are two basic strategies to implement the biological control of weeds: the introduction of foreign pathogenic organisms, called the “classical approach,” and the “augmentative” or “bioherbicidal approach,” where the pathogenic organisms are already present (native or introduced) and their population is increased by mass rearing. In epidemiological terms, these approaches are described as “inoculative” and “inundative strategy,” respectively [39].

#### 4.4.1. Classical Strategy

The “inoculative” or “classical approach” implies the control of invasive weeds by introduction of suitable, exotic bioagent from the weed's natural habitat. The main objective of classical biological weed control is restoring balance between target alien weed and its natural enemies in the ecosystem. Successful bioagent reduces the weed population first then the bioagent population dies due to starvation of food. This process continues in cyclic fashion until the bioagent and weed population get established at a low level. A successful control strongly depends on favourable conditions for the bioagent, which effectively increase the population of the controlling organism [40]. This method is a slow operation and currently used in noncropped areas. The control of different weeds through the use of classical biological agents, insects, and fungal plant pathogen is given in Table 2 [30, 41].

#### 4.4.2. Bioherbicidal Approach

“Plant pathogenic fungi are developed and used in the inundative strategy to control weeds in the way chemical herbicides are used,” or as “living products that control specific weeds in agriculture as effectively as chemicals” [42]. Usually, they are applied in a manner similar to chemical herbicides (hence called bioherbicides) by periodic dispersals of distinct doses of the virulent inoculum [37, 43]. The concept of mycoherbicides was introduced by Daniel et al. [44], who demonstrated that an endemic pathogen might be rendered completely destructive to its weedy host by applying a massive dose of inoculum at a particularly susceptible growth stage. To achieve success, the pathogen must be culturable in artificial media; the inoculum must be capable of abundant production using conventional methods such as liquid fermentation; the final product must be genetically stable and specific to the target weed; storage (shelf-life), handling, and methods of application must be compatible with current agricultural practices; and the pathogen must be efficacious under sufficient different environment conditions to allow a feasible application window [44]. In the past, several attempts have been made to control weeds with fungal products or mycoherbicides [38] and several products of mycoherbicides are available in the market (Table 3) and many more are in the pipeline.

## 5. Biological Control of Parthenium

### 5.1. Classical Biological Control

**Figure 4:**
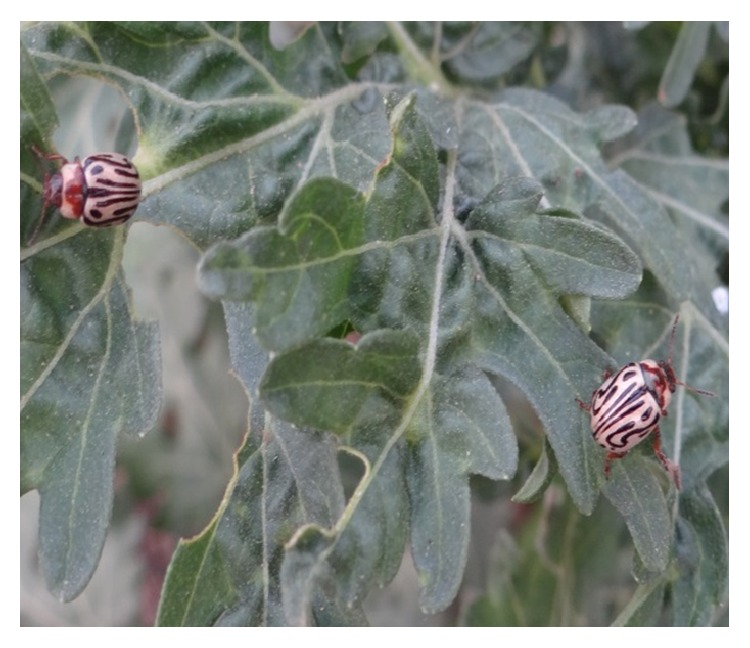
*Zygogramma bicolorata* feeding on parthenium weed.

(a)* Insects as Classical Biocontrol Agents.* Several insects have been tried to control parthenium weed in the different countries (Table 4). Of the various insects, the leaf-feeding beetle (*Zygogramma bicolorata*) (Figure 4) and the stem galling moth (*Epiblema strenuana*), both imported from Mexico, have shown good potential to control this weed. The beetle,* Z. bicolorata*, an effective leaf eater, was imported from Mexico for the management of parthenium in Australia in 1980, and in Indian Institute of Horticulture Research (IIHR) [45]. Both the adults and larvae of this insect feed on leaves. The early stage larvae feed on the terminal and auxiliary buds and move on to the leaf blades as they grow. The fully-grown larvae enter the soil and pupate. An insect density of one adult per plant caused skeletonization of leaves within 4–8 weeks but little success has been achieved as the weed has very high generative potential, and moreover the insect is not a species specific and is found to attack sunflower in India [41]. Attempts have also been made to introduce* Epiblema strenuana*—a stem galling moth, but as this moth lays eggs and develops on niger crops [45], so its cultures were destroyed.

(b)* Classical Control by Fungal Plant Pathogens.* In standard classical biological control strategy, obligate parasites, especially rust fungi, are the first choice because they exhibit narrow host ranges, high reproductive capacities, and efficient aerial dispersal [46]. The most promising fungal agents to manage parthenium are* Puccinia abrupta* var.* partheniicola* (Jackson)* Parmelee*,* Puccinia xanthii* var.* parthenii-hysterophorae* (previously known as* P. melampodii Diet.* and* Holw.*) (Uredinales)*, Entyloma compositarum De Bary* (Ustilaginales), and* Plasmopara halstedii* (Farlow)* Berl. and De Toni* (Peronosporales). Of these,* Puccinia abrupta* var.* partheniicola* and* Puccinia xanthii* var.* parthenii-hysterophorae* originate from Mexico and have been fully screened and released in Australia; they are the most potential classical biocontrol fungal pathogens of this weed in Australia.

#### 5.1.1. *Puccinia abrupta* var.* partheniicola*

A rust pathogen,* Puccinia abrupta *var.* partheniicola*, indigenous to Mexico, was introduced in 1999 to Australia to control parthenium, as a classical biocontrol agent. The rust is commonly found in high to mid altitude (1400–2500 m.a.s.l.) with disease incidence up to 100% in some locations. The incidence of the rust disease on parthenium in different locations under field conditions showed varied effects on morphological parameters of this weed, with seed production capacity reduced by 42%. Host specificity tests against the weed and crop hosts related to parthenium revealed that sporulation of* P. abrupta* were observed exclusively on parthenium, though limited number of poorly developed pustules were recorded on varieties of niger seeds (*Guizotia abyssinica*) [47, 48].

#### 5.1.2. *Puccinia xanthii* var.* parthenii-hysterophorae*

*Puccinia xanthii *var.* parthenium-hysterophorus* is an autoecious, microcyclic rust fungus, producing both telia and basidiospores on one host [49]. The teliospores germinate over a wide temperature range (optimum being 25°C) and produce basidiospores (optimum at 22°C), which directly penetrate the host epidermis [50, 51]. Prior to the release of* P. xanthii *var.* parthenii-hysterophorae* in Australia and South Africa, host-specificity testing was conducted on over different plant species within the family Asteraceae. Limited infection was observed on a few plants, but in most cases the infection consisted of abnormal or abortive sporulation, and the level of sporulation was much less than those on parthenium weed [50, 52]. Since its release in Australia in 2000 there have been no reports of* P. xanthii *var.* parthenii-hysterophorae* infecting any plant other than* Parthenium hysterophorus,* and it is thought to be a promising pathogen for controlling this weed in Australia. It is expected that* P. xanthii *var.* parthenii-hysterophorae* will also contribute greatly to the management of parthenium weed infestations in the warm, lower-altitude regions of South Africa, where there are currently no biological control agents implemented against this weed.

### 5.2. Inundative Biological Control

A series of surveys have been carried out to search for naturally occurring fungal pathogens on parthenium to control it through the bioherbicidal strategy. The pathogens reported on parthenium from world are listed in Table 5.

There is a long list of fungal pathogens recorded on parthenium around the globe, out of which six have been evaluated for their biocontrol potential which are discussed here.

Saxena and Kumar [53] worked on the mycoherbicidal potential of* Alternaria alternata *ITCC (LC#508) in northern India to control parthenium weed and reported 50% damage of plants* in vitro* detached leaf and whole plant bioassay at 96 hours after treatment at a concentration of 1 × 10^6^ spores/mL.* Sclerotium rolfsii *(teleomorph:* Athelia rolfsii*) incites a severe collar rot disease on parthenium [54, 55]. Although, the pathogen is responsible for severe damage to the weed, but the wide host range of the species creates doubt about its suitability as mycoherbicides.* Cercospora parthenophilia, *a leaf spot pathogen isolated from parthenium at Kurukshetra, has shown several characteristics that make it a potential biological control agent of this weed in India such as wide natural distribution; it sporulates well on Czapek dox agar (a simple and cheap culture medium), within ten days, and can thus be mass produced in a short time and at low cost; it has narrow host range and capable of limiting populations of the weed under experimental conditions [56].* Cladosporium *sp. (MCPL-461), a floral leaf pathogen of parthenium, produces symptoms on the flowers, buds, and inflorescences, and causes sterility and reduces seed viability. The severity of pathogen to the reproductive organs led to serious damages of the partheniumplants and may be used as a potential mycoherbicide against this weed [5]. Satyaprasad and Usharani [57] reported powdery mildew causing* Oidium parthenii* on parthenium at Hyderabad. The fungus appears as small, circular, white powdery spots on the surface of leaves and spreads over the entire lamina on both the surfaces giving a powdery appearance to the plant. Severe infection leads to defoliation. Kauraw et al. [58] reported* Fusarium pallidoroseum,* on parthenium from Jabalpur. It was found to reduce seed germination, seedling vigour, height of plant, number of branches, and number of flowers and reported as a potential biocontrol agent for parthenium management.

### 5.3. Integrated Weed Management

The classical and bioherbicidal strategies, when applied alone, are not able to suppress this weed. However, integrated pest management (IPM) has gained attention in recent years as a means of reducing losses due to pests, minimizing reliance on chemical pest control, therefore fostering the long-term sustainability of agricultural systems. In Australia, to complement the classical biological control approach with other management tactics, two selected suppressive plants, the native Mitchell grass (*Astrebella squrossa*) and the introduced legume, butterfly pea (*Clitoria ternatea*) along with two biological control agents, a leaf and a seed feeding beetle (*Zygogramma bicolorata*) and a stem galling moth (*Epiblema strenuana*), have been used to control parthenium weed under integrated weed management. The suppressive plants significantly suppressed weed growth in the absence of the biological control agents. However, this suppressive ability could be further enhanced in the presence of one of the either aforementioned biological agents. Work carried out in Australia has revealed that the parthenium weed can be more effectively managed by complementing presently existing biological control strategies with suppressive plants [52]. Shabbir [59] conducted another experiment in Australia for a two-year period to control parthenium weed. They used six suppressive plant species with biological control agents (*Epiblema strenuana* Walker,* Zygogramma bicolorata Pallister*,* Listronotus setosipennis* Hustache, and* Puccinia abrupta* var.* partheniicola*) in the field to reduce the growth of parthenium weed between 60–80% and 47–91% in the years 1 and 2, respectively. The biomass of the suppressive plants was between 6% and 23% greater when biological control agents were present than when the biological control agents had been excluded. This shows that parthenium weed can be more effectively managed by combining the current biological control management strategy with selected sown suppressive plant species.

## 6. Conclusions

The noxious* P. hysterophorus* grows in a wide variety of habitats and causes changes in above ground vegetation as well as in below ground soil nutrients. It is capable of out-competing native and nonnative palatable plants that are important to livestock. Furthermore, the changes in vegetation and soil nutrients could lead to ultimate changes in other trophic levels and alter the function of the ecosystem. Appropriate methods for the management of* P. hysterophorus* are necessary to avoid potential threats to biodiversity and economic losses. The efficient and environment-friendly alternative to other time-consuming, costly, toxic, physical, and chemical methods is the use of biological control through allelopathy, insects and fungal pathogens. Nine insect species and two rusts have been released in Australia to check this weed. Of these, two insects* Z. bicolorata* and* E. stenuana,* and two rust fungi,* Puccinia abrupta *var.* partheniicola* and* Puccinia xanthii *var.* parthenii-hysterophorae*, have shown potential and are being used to control this weed. Nevertheless the weed has not been completely checked and is still creating nuisance in both Australia and India, and more needs to be done by scientists, agriculturists, and government to work jointly for managing this troublesome weed.

## Figures and Tables

**Table 1:** Parthenium weed control at rosette and bolted stages with different herbicidal application at 4 weeks after treatment (WAT).

Serial number	Herbicides	% Mortality at rosette stage	% Mortality at bolted stage
1	Glyphosate	96	91
2	Metribuzin	87	75
3	2,4-D	71–80	43
4	Bromoxynil + MCPA	57–79	50–61.5
5	Atrazine	56.5	36.5
6	S-metolachor	57.5	41
7	Pendimethalin	42.5	30

**Table 2:** Successful examples of control of weeds through classical biocontrol agents.

Weed	Bioagent	Kind of bioagent	Reporting country
*Chondrilla juncea *	*Puccina chondrillina *	Rust	Australia
*Cyperus rotundus *	*Bactra verutana *	Shoot boring moth	India, Pakistan, USA
*Eupatorium riparium *	*Entyloma compositarum *	Plant pathogen	USA
*Hydrilla verticillata *	*Hydrellia pakistanae *	Shoot fly	USA
*Orobanche cernua *	*Sclerotinia *sp.	Plant pathogen	USA
*Parthenium hysterophorus *	*Puccinia abrupta *var. *partheniicola *	Rust	Mexico
*Parthenium hysterophorus *	*Zygogramma Bicolorata * *Epiblema strenuana * *Conotrachels* sp.	Leaf eating bettle, Stem galling insect, Stem galling insect	Mexico Australia Australia
*Rumex *spp.	*Uromyces rumicis* * Gastrophysa viridula *	Plant pathogen Beetle	USA USA
*Tribulus terrestris *	*Microlarinus lareynii *and* M. lypriformis *	Pod weevil	USA

**Table 3:** Examples of weed control using bioherbicidal approach (liquid and solid formulations).

Serial number	Target weed	Fungus	Product name	Year of registration	Formulation type
Liquid formulations					
1.	Persimmon (*Diospyros virginiana*) trees in rangelands	*Acremonium diospyri *	*Acremonium diospyri *	1960	Conidial suspension
2.	Dodder (*Cuscuta chinesis* and *C. australis*) in soybeans	*Colletotrichum gloeosporioides *f. sp. *Cuscutae *	Lubao	1963	Conidial suspension
3.	Milkweed vine (*Morrenia odorata*)	*Phytophthora palmivora *(*P. citrophthora*)	DeVine	1981	Liquid spores suspension
4.	Yellow nutsedge (*Cyperus esculentus*)	*Puccinia canaliculata *	Dr. Biosedge	1987	Emulsified suspension
5.	Turf grass (*Poa annua*) in golf courses	*Cylindrobasidium leave *	Stumpout	1997	Liquid (oil) suspension
6.	Woody plants Blackberry weed (*Prunus serotina*)	*Chondrostereum purpureum *	BioChon	1997	Mycelial suspension in water
7.	*Hakea gummosis* and *H. sericea *in native vegetation	C*olletotrichum acutatum *	Hakatak	1999	Conidial suspension
8.	Deciduous tree species	*Chondrostereum purpureum *	Mycotech Paste	2004	Paste
9.	Alder, aspen, and other hardwoods	*Chondrostereum purpureum *	Chontrol (Ecoclear)	2004	Spray emulsion and paste
10.	Dodder species	*Alternaria destruens *	Smolder	2008	Conidial suspension
11.	Soda apple (*Solanum viarum) *	Tobacco mild green mosaic virus	Solvinix	2009	Foliar spray suspension

Solid formulations					
1.	Northern joint-vetch (*Aeschynomene virginica*)	*Colletotrichum gloeosporioides *f. sp. *aeschynomene *	Collego LockDown	1982	Wettable powder
2.	Sickle-pod and coffee senna (*Cassia* spp.)	*Alternaria cassia *	Casst	1983	Solid
3.	Water hyacinth (*Eichhornia crassipes*)	*Cercospora rodmanii *	ABG-5003	1984	Wettable powder
4.	Velvet leaf (*Abutilon theophrastus*)	*Colletotrichum coccodes *	Velgo	1987	Wettable powder
5.	Round-leaved mallow (*Malva pussila*)	*Colletotrichum gloeosporioides *f. sp. *malvae *	BioMal	1992	Mallet wettable powder
6.	*Hakea gummosis* and *H. sericea *in native vegetation	C*olletotrichum acutatum *	Hakatak	1999	Granular (dry conidia)
7.	Dyers woad (*Isastis tinctoria*) in farms, rangeland, waste areas, and roadsides	*Puccinia thlaspeos *	Woad Warrior	2002	Powder
8.	Dandelion (*Taraxacum officinale*) in lawns/turf	*Sclerotinia minor *	Sarritor	2007	Granular

**Table 4:** Insect biocontrol agents released to control parthenium weed in different countries.

Biological control agent	Feeding habits	Native country	Released country
*Bucculatrix parthenica *	(Leaf mining moth)	Mexico	Australia
*Conotrachelus albocinereus *	(Stem galling weevil)	Mexico	Australia
*Epiblema strenuana *	(Stem galling moth)	Mexico	Australia
*Listronotus setosipennis *	(Stem boring weevil)	Argentina and Brazil	Australia
*Platphalonidia mystica *	(Stem boring moth)	Argentina	Sri Lanka
*Smicronyx lutulentus *	(Seed feeding weevil)	Mexico	Pakistan, Australia
*Stobaera concinna *	(Parthenium sap feeder plant hopper)	Mexico	Australia
*Zygogramma bicolorata *	(Leaf feeding beetle)	Mexico	Australia, India

**Table 5:** Fungal pathogens recorded on parthenium around the world [5, 60].

Fungus	Countries	References
*Alternaria alternata *	India	[61]
*Alternaria alternata * ITCC (LC#508)	India	[53]
*Alternaria protenta *	Mexico	[62]
*Alternaria zinniae *	Mexico, India	[63]
*Cercospora parthenii *	Cuba, Mexico, India	[64]
*Colletotrichum dematium *	India	[65]
*Colletotrichum capsici *	India	[66]
*Colletotrichum gloeosporioide *	India	[67]
*Cladosporium *sp. (MCPL-461)	India	[5]
*Curvularia lunata *	India	[68]
*Curvularia palesens *	India	[69]
*Curvularia verruculsa *	India	[69]
*Dreshlera australiensis *	India	[69]
*Dreshlera hawaiiensis *	India	[69]
*Erysiphe cichoracearum *	India	[56]
*Exerohilum rostratum *	Mexico	[70]
*Fusarium semitectum *	Mexico	[70]
*Fusarium oxysporum *	India	[71]
*Fusarium pallidoroseum *	India	[58]
*Fusarium solani *	India	[71]
*Lasiodiplodia theobromae *	India	[72]
*Myrothecium roridum *	India	[73]
*Oidium parthenii *	India	[57]
*Phoma sorghina *	India	[74]
*Rhizoctonia solani *	India	[75]
*Sclerotinia sclerotiorum *	India	[76, 77]
*Sclerotium rolfsii *	India	[78]
*Sphaerotheca fuliginea *	India	[79]
*Syncephalastrum racemosum *	India	[69]
